# *In Vitro* and *In Vivo* Activity of a Novel Antifungal Small Molecule against *Candida* Infections

**DOI:** 10.1371/journal.pone.0085836

**Published:** 2014-01-22

**Authors:** Sarah Sze Wah Wong, Richard Yi Tsun Kao, Kwok Yong Yuen, Yu Wang, Dan Yang, Lakshman Perera Samaranayake, Chaminda Jayampath Seneviratne

**Affiliations:** 1 Faculty of Dentistry, University of Hong Kong, Hong Kong; 2 Department of Microbiology, University of Hong Kong, Hong Kong; 3 Department of Pharmacology & Pharmacy, Li Ka Shing Faculty of Medicine, University of Hong Kong, Hong Kong; 4 Department of Chemistry, Faculty of Science, University of Hong Kong, Hong Kong; Worcester Polytechnic Institute, United States of America

## Abstract

*Candida* is the most common fungal pathogen of humans worldwide and has become a major clinical problem because of the growing number of immunocompromised patients, who are susceptible to infection. Moreover, the number of available antifungals is limited, and antifungal-resistant *Candida* strains are emerging. New and effective antifungals are therefore urgently needed. Here, we discovered a small molecule with activity against *Candida* spp. both *in vitro* and *in vivo*. We screened a library of 50,240 small molecules for inhibitors of yeast-to-hypha transition, a major virulence attribute of *Candida albicans*. This screening identified 20 active compounds. Further examination of the *in vitro* antifungal and anti-biofilm properties of these compounds, using a range of *Candida* spp., led to the discovery of SM21, a highly potent antifungal molecule (minimum inhibitory concentration (MIC) 0.2 – 1.6 µg/ml). *In vitro*, SM21 was toxic to fungi but not to various human cell lines or bacterial species and was active against *Candida* isolates that are resistant to existing antifungal agents. Moreover, SM21 was relatively more effective against biofilms of *Candida* spp. than the current antifungal agents. *In vivo*, SM21 prevented the death of mice in a systemic candidiasis model and was also more effective than the common antifungal nystatin at reducing the extent of tongue lesions in a mouse model of oral candidiasis. Propidium iodide uptake assay showed that SM21 affected the integrity of the cell membrane. Taken together, our results indicate that SM21 has the potential to be developed as a novel antifungal agent for clinical use.

## Introduction

*Candida* is the most common fungal pathogen of humans and the fourth leading pathogen in nosocomial bloodstream infections [1]. It causes a wide range of infections, from invasive to superficial, at various anatomical sites [2]. Invasive candidiasis is associated with high mortality among immunocompromised populations, and superficial mucosal candidiases are highly prevalent and persistent, especially among immunocompromised patients. The most common superficial candidiases are oral candidiasis and *Candida*-associated denture stomatitis [3]–[5]. *Candida*-associated denture stomatitis could affect up to 70% of denture wearers [6].

The most prevalent candidiasis-causing species is *Candida albicans*. It has a versatile morphogenesis, existing in three forms (*i.e.*, yeasts, pseudohyphae and hyphae), and has two lifestyles (planktonic and biofilm), permitting *C. albicans* to thrive under diverse environmental conditions. In particular, the yeast-to-hypha (Y-H) transition is a major virulence attribute that facilitates tissue invasion by *C. albicans* [7], [8]. In addition to Y-H transition, biofilm formation on host tissue or abiotic devices is also a significant basis for *Candida* infections [5], examples include *Candida*-associated denture stomatitis [9]. The formation of *Candida* biofilms, which are more resistant to antifungal agents than planktonic cells, is directly associated with treatment failure [5], [10]. Although *Candida* infections have always posed a heavy burden on public health, this situation has recently worsened. First, the worldwide incidence of candidiasis has been increasing over the past few decades [11], which may be attributable to increased numbers of immunocompromised patients and the use of broad-spectrum antibiotics [12]. Second, the increased incidence of invasive candidiasis caused by non-*albicans Candida* (NAC) species, such as *Candida glabrata*, *Candida tropicalis*, *Candida krusei* or *Candida parapsilosis*, has been a major concern [13], [14], because these infections are often associated with higher mortality and antifungal resistance than those caused by *C. albicans* [15], [16]. Lastly, the emergence of antifungal resistance and treatment side effects have further restricted treatment options because of the already limited arsenal of current antifungal agents [17], [18].

Only a few classes of antifungal drugs (polyenes, azoles, echinocandins, allylamines and DNA analogues) are available for candidiasis treatment [19], but these drugs are far from ideal. Polyenes, for example, have dose-related toxicity, particularly nephrotoxicity (even though the recent introduction of lipid formulations has now improved the risk-benefit ratio) [20]. In addition, rising drug resistance is an inevitable problem. This is pertinent for fluconazole, a drug of choice for treating AIDS patients with *Candida* infections. While *C. glabrata* and *C. krusei* are intrinsically resistant to fluconazole, the emergence of fluconazole-resistant *C. albicans* strains is also on the rise [21], [22]. Similarly, emerging resistance has also been reported for the more recently introduced echinocandins [23]–[26]. Therefore, the situation is likely to be ameliorated only by the discovery and introduction of safe and new antifungal agents.

**Figure 1:**
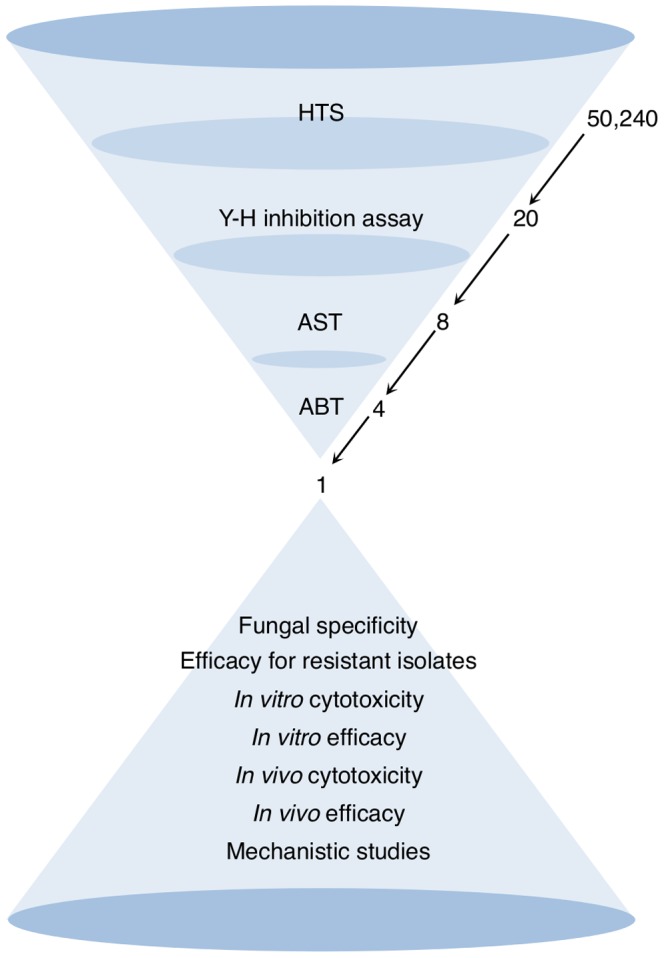
Strategy for screening for novel antifungal small molecules. We screened for Y-H inhibitors in a library containing 50,240 small molecules and found 20 active compounds. These 20 primary hits were further validated by assessing their activity in a dose-dependent manner, which led to the identification of eight potent Y-H inhibitors. The antifungal properties of the eight compounds were analysed in antifungal susceptibility tests, and the four most potent molecules were selected. Finally, the anti-biofilm activity of the four hits was evaluated, and SM21 was chosen for comprehensive *in vitro* and *in vivo* assays. HTS, high-throughput screening; AST, antifungal susceptibility test; ABT, anti-biofilm test.

Small molecules are an invaluable source of novel antifungal agents [27]. Previous screening of the same small-molecule library has resulted in the identification of novel compounds effective for severe acute respiratory syndrome-associated coronavirus [28]. Here, we sought small molecules with anti-*Candida* properties (with the overall strategy depicted in Figure 1). We first screened a collection of over 50,000 small molecules for inhibitors of the Y-H transition in *C. albicans*. The identified hits were further assessed for antifungal and anti-biofilm activity, which led us to discover a novel antifungal small molecule, which we designated SM21. We examined the *in vitro* antifungal activity of SM21, as well as its *in vivo* efficacy in mouse infection models of oral and systemic candidiases. Last, the potential mechanism of action of SM21 was also investigated.

## Materials and Methods

### High-throughput screening for Candida Y-H inhibitors

High-throughput screening was performed at the Chemical Genetics Unit, Department of Microbiology, Research Center of Infection and Immunology, Li Ka Shing Faculty of Medicine, University of Hong Kong, on a library with 50,240 small molecules (ChemBridge, San Diego, CA, USA) to identify inhibitors of Y-H transition in *C. albicans* SC5314, as was previously described [29]. *C. albicans* SC5314 were seeded at 5×10^3^ cells per well in complete yeast peptone dextrose (YPD) (1% yeast extract, 2% peptone, 2% glucose/dextrose) supplemented with 20% heat-inactivated foetal bovine serum (Invitrogen, Carlsbad, CA, USA) in a total volume of 50 µl in 384-well microtitre plates. The small molecules were dissolved in dimethyl sulfoxide (DMSO) and were added to the wells at final concentration of 20 µg/ml, whereas the controls contained the same amount of DMSO but without small molecules. Assay plates were incubated at 37°C in 5% CO_2_ for 12 h. Morphologies of the *Candida* were scored using a Leica DMIL inverted microscope equipped with DC300F digital imaging system (Leica Microsystems, Heidelberg, Germany). Small molecules with lower scores than that of the control (yeast-to-hypha transition inhibitors) were selected as primary hits.

### Further evaluation of Y-H inhibition of the primary hits

Dose-dependent Y-H inhibition by the primary hits was further examined using the reference strain *C. albicans* SC5314 and ten clinical isolates of *C. albicans* (strains CL1 – CL10, of oral origin from the archival collection of Oral Biosciences, Faculty of Dentistry, University of Hong Kong) under robust hypha-inducing conditions (Lee's medium [30] and 37°C incubation). Each sample contained 100 µl of *Candida* suspension (1×10^6^ CFUs/ml) and 100 µl of the small molecules of different final concentrations (3.13 – 25 µg/ml). After incubation for 24 h, cell morphologies were observed by light microscopy (Olympus BH-2, Tokyo, Japan). The degree of Y-H inhibition was quantified by calculating the percentage of hyphal cells in one hundred cells in a single sample [29]. Counting was performed in quadruplicate for each sample. The minimum inhibitory concentration (MIC) of Y-H transition (MIC_Y-H_) was determined as the minimum concentration at which no hyphae were observed. The assay was performed on three different occasions in duplicate.

### Antifungal susceptibility tests of the small molecules

The antifungal activity of small molecules was assessed by disk diffusion and broth dilution assays (see below) using four groups of *Candida* isolates. The first group consisted of ATCC strains: *C. albicans* ATCC 90028 (quality control strain), *C. glabrata* ATCC 90030, *C. krusei* ATCC 6258, *C. parapsilosis* ATCC 22019 and *C. tropicalis* ATCC 13803.

The other three groups were clinical isolates: 92 bloodstream isolates (Queen Mary Hospital and Queen Elizabeth Hospital, Hong Kong), 46 oral isolates from nasopharyngeal carcinoma patients (Queen Mary Hospital, Hong Kong) and 10 denture stomatitis clinical isolates (from the archival collection of Oral Biosciences, Faculty of Dentistry, University of Hong Kong). All of the isolates were subcultured on Sabouraud dextrose agar (SDA) and were incubated aerobically at 37°C overnight before the assay. All of the assays were performed on three different occasions in duplicate per isolate.

### Disk diffusion assay

The standard disk diffusion assay (Clinical and Laboratory Standards Institute (CLSI) M44-A2) was used. In brief, a 1×10^6^ CFUs/ml yeast suspension was prepared for each isolate in phosphate-buffered saline (PBS) and distributed evenly by a spiral plater on Mueller-Hinton (M-H) agar supplemented with 2% glucose and 0.5 µg/ml methylene blue. Antifungal tablets (10 µg amphotericin B, 5 µg caspofungin, 15 µg ketoconazole and 25 µg fluconazole) (Neo-Sensitabs, Rosco Diagnostica, Taastrup, Denmark) were used as positive controls. Paper coupons were placed on the agar, and either 2 µg of the small molecule or PBS (as a negative control) was added onto the coupons. The M-H agar plates were then incubated aerobically at 37°C for 24 h, and diameters of the resultant inhibition zones were measured.

### Broth dilution assay

The standard protocol for broth microdilution assay (CLSI M27-A3) was followed with modifications [31]. Yeast suspensions (approximately 1×10^3^ colony forming units per millilitre (CFUs/ml)) were prepared in RPMI 1640 medium (Life Technologies, New York, USA) and were added to a 96-well microtitre plate (Iwaki, Tokyo, Japan). Serial dilutions of the small molecules were prepared with the medium and added to the wells. Amphotericin B was used as a positive control. The plate was incubated at 37°C for 48 h, and then *Candida* growth on the well bottoms was visually observed to determine the minimum inhibitory concentration (MIC). Activity against *Cryptococcus neoformans*, *Aspergillus fumigatus* and *Penicillium marneffei* was similarly assessed after 72 h of incubation.

### Anti-biofilm assay in microtitre plates

*Candida* biofilms were formed according to a previously described method [32]. In brief, yeast cells were subcultured on SDA and incubated at 37°C aerobically for 18 h. They were then transferred to yeast nitrogen base (YNB) liquid medium supplemented with 50 mM glucose, and incubated aerobically at 37°C, 75 rpm overnight. The harvested cells were washed twice with 10 ml PBS and were resuspended to a density of 1×10^7^ CFUs/ml in YNB medium supplemented with 100 mM glucose. Each of the wells of the microtitre plate (Iwaki) was inoculated with 100 µl yeast suspension. The cells were allowed to adhere to the well bottoms by incubating at 37°C, 75 rpm for 1.5 h (adhesion phase). Then, the biofilm was washed with 100 µl PBS to remove the non-adherent cells, and 200 µl fresh medium was added to each well. The biofilm was then incubated at 37°C, 75 rpm for 24 h. For the screening assay, the small molecules (0.2 – 100 µg/ml) were added to the biofilm before or after the adhesion phase. The effect of SM21 on mature biofilms was further investigated by adding SM21 (0.2 – 100 µg/ml) to the biofilm at 24 h and 48 h.

Cell viability of the treated biofilms was measured by the XTT reduction assay [33]. The medium in each well was discarded, and 200 µl XTT solution was added. The XTT solution consisted of 40 µl XTT stock solution (1 mg/ml in PBS), 2 µl menadione (0.4 mM in acetone) and 158 µl PBS. The microtitre plate was incubated at 37°C in the dark for 3 h. Then, 160 µl XTT solution from each well was transferred to a new microtitre plate, and the absorbance was measured at 490 nm. The minimum inhibitory concentration for biofilm (MIC_biofilm_) was determined as the concentration that caused a 50% reduction in cell viability. All of the assays were performed on three different occasions in duplicate.

### Anti-biofilm assay using an in vitro denture stomatitis model

Cold-cured polymethylmethacrylate (PMMA) discs were prepared according to the manufacturers' instructions (Vertex RS, Vertex-Dental BV, Zeist, The Netherlands) and as described previously with some modifications [34]. In brief, transparent self-polymerising acrylic powder was mixed with monomer liquid at a 1.5∶1 ratio (w/v). This mixture was then immediately spread onto and pressed tightly between two aluminium foil-covered glass slides that had been secured with two binder clips to ensure similar thickness and surface of the resultant acrylic strips. After curing for 15 min at 40°C, 2 – 6 bar, the strips were carefully retrieved and cut into 1-cm^2^ squares. The strips were immersed in distilled water for 7 days to remove excess monomers, and then disinfected in 70% alcohol for 1 min and washed thrice with sterile distilled water. To simulate conditions present in the mouth and to promote *Candida* adhesion, the strips were treated with saliva for 30 min at 37°C [35]. The saliva had been collected from five healthy volunteers and centrifuged at 12,000× *g* for 15 min at 4°C, and the resulting supernatant was stored at −70°C until use [36].

*C. albicans* isolate BF-1, which was identified as a stronger biofilm former in a previous study [36], was subcultured on SDA one day before the assay. The yeast inoculum was standardised to 1×10^7^ CFUs/ml in YNB supplemented with 100 mM glucose.

The acrylic strips were placed in 24-well plates (Iwaki), and 1 ml yeast inoculum was added to each well, completely covering the acrylic strips. SM21 was added at MIC_biofilm_ (1.56 µg/ml) before, after, or both before and after the adhesion phase. The adhesion phase was assessed 1.5 h after inoculation, and the plate was then placed in a shaking incubator at 37°C. The cell viability of the biofilm on the acrylic strips was quantified by the XTT reduction assay and was also observed by confocal laser scanning microscopy (Olympus Fluoview FV1000) after staining using a LIVE/DEAD BacLight Viability Kit (Invitrogen).

### Analysis of HWP1 expression

Engineered *C. albicans* strain P*_HWP1_*-*GFP*, which expressed GFP under the control of either the *HWP1* promoter, was gift from Dr. B. P. Krom [37]. The P*_HWP1_*-*GFP* strain was used to test the effect of SM21 on *HWP1* expression. The strains were cultivated overnight at 37°C in Lee's medium containing SM21 at MIC (0.2 µg/ml) or 2×MIC (0.4 µg/ml). GFP expression was then observed using confocal laser scanning microscopy (Olympus Fluoview FV1000).

### Fungal specificity assay

The disk diffusion assay (CLSI M44-A2) was also used to assess the potential activity of SM21 against six bacterial species (*Escherichia coli, Lactobacillus acidophilus*, *Streptococcus mutans*, *Streptococcus mitis, Streptococcus sanguinis* and *Aggregatibacter actinomycetemcomitans*). The bacteria were subcultured one day before the assay. Bacterial cell suspensions were prepared for each sample at a density of 1×10^6^ CFUs/ml and were then distributed evenly on sheep blood agar plates with a spiral plater. Paper coupons inoculated with 10 µl of drugs or controls (0.2 µg/µl SM21, 1 µg/µl amphotericin B and 0.5 µg/µl caspofungin) were placed on the agar. Chlorhexidine gluconate (0.2%) was used as a positive (antibacterial) control, and PBS as a negative (harmless) control. The plates were incubated anaerobically (except for the *E. coli* plate) at 37°C for 24 h, and the diameters of inhibition zones were measured.

### Effects on antifungal-resistant isolates

The efficacy of SM21 against 16 antifungal-resistant *Candida* strains was evaluated by antifungal susceptibility tests (broth microdilution and disk diffusion assays) as described above. The strains were clinical blood isolates belonging to various *Candida* spp., which had been obtained from Helsinki University Central Hospital (Helsinki, Finland) and that had been confirmed to have resistance to one or more antifungal agents by standard CLSI antifungal susceptibility tests in our previous study [25].

### Cytotoxicity assays

Primary human oral keratinocytes (HOKs) (ScienCell Research Laboratories, Carlsbad, CA, USA) at passage three were seeded into a 96-well plate (Iwaki) in oral keratinocyte medium (ScienCell Research Laboratories). The serum-free medium contained 1% oral keratinocyte growth supplement, 1% penicillin and 1% streptomycin. Each well initially contained approximately 1×10^4^ HOKs, which were incubated in the presence of 5% CO_2_ at 37°C (medium was changed every day) until the cells reached confluence. A dilution series of SM21 (0.05 – 54.8 µg/ml) was then supplied to the wells so that each well contained 200 µl of medium in total. The plate was incubated at 37°C for 24 h, and then the viability of the HOKs was determined by MTT assay (see below).

T-75 flasks were also used to assess cell viability in the presence of SM21. Besides HOKs, human gingival fibroblasts (HGFs) and monocyte cell lines (ScienCell Research Laboratories) were also used. Each cell line was seeded in T-75 flasks, and after confluence was reached, 2 µg SM21 in 10 ml of growth medium (0.2 µg/ml final concentration) was added to each flask. For comparison, amphotericin B was used at the same concentration. After 24 h-incubation at 37°C, MTT assay was performed to measure the cell viability.

MTT (3-(4,5-Dimethylthiazol-2-yl)-2,5-diphenyltetrazolium bromide) is a yellow tetrazolium dye that is converted into a purple compound by mitochondrial enzymes. MTT solution (5 mg/ml) was prepared in growth medium and added to the wells or flasks. After incubation at 37°C for 3 h in dark, the MTT solution was discarded. The converted dye was solubilised with an equal amount of DMSO. The absorbance of the converted dye was measured at 570 nm with background subtraction at 650 nm. The concentration at which the cell viability had dropped by 50% was recorded as the cytotoxic concentration (CC_50_) [38].

### Co-culture of *C. albicans* and HOKs

HOK culture was performed as mentioned above. HOKs at passage three were seeded into an 8-well plate (ibidi, Martinsried, Germany), which was compatible for the observation with confocal laser scanning microscope. The cells were incubated at 37°C in the presence of 5% CO_2_ until confluence was reached, with the medium changed every day. Each well contained approximately 1×10^5^ HOKs at confluence. The cells were washed once with PBS, and fresh medium without antibiotics (as antibiotics could inhibit *Candida* growth) was added. A suspension of *C. albicans* SC5314 (1×10^4^ CFUs/ml obtained by subculturing on SDA plates and incubating at 37°C one day before inoculation) was prepared in the growth medium (without antibiotics), and 100 µl of this cell suspension was added to each well. SM21 was added at various final concentrations (0.5, 1 and 2 µg/ml), whereas the positive controls contained only the growth medium. The plate was then incubated at 37°C for 24 h in the presence of 5% CO_2_. The viability of HOKs and yeast cells was assessed by confocal laser scanning microscopy using fluorescent probes (LIVE/DEAD BacLight Viability Kit and LIVE/DEAD Viability/Cytotoxicity Kit for mammalian cells; Invitrogen).

### Ethics Statement

The animal studies strictly followed the recommendations in “Guide for the Care and Use of Laboratory Animals” published by National Institutes of Health. The protocols were approved by the Committee on the Use of Live Animals in Teaching and Research (CULATR), University of Hong Kong (Permit Number: 2461-11).

### Mouse model of systemic candidiasis

The mice used were 8-week-old male C57BL/6 mice that were obtained from the Laboratory Animal Unit, University of Hong Kong. Mouse model of systemic candidiasis was established according to a previously described method [39], with modifications. The mice were injected via the tail vein with 100 µl cell suspension of *C. albicans* SC5314 (1×10^6^ CFUs/ml) 3 h before the start of antifungal treatment. Drugs were given to the mice twice a day for 5 days via intraperitoneal injection. The test group (n = 5) was treated with 0.01, 0.1, 1 and 10 mg/kg SM21 in 100 µl PBS, while the control group (n = 5) was treated with PBS. The mice were weighed every day. All of the mice were sacrificed after 5 days, and the kidneys were harvested (as most of the *Candida* cells in systemic candidiasis murine models are found in the kidneys [39]). The kidneys were divided into equal portions for fungal burden determination and histopathological evaluation. For fungal burden determination, the kidneys were weighed and then homogenised in PBS, and the homogenates were serially diluted before plating on SDA. The plates were incubated at 37°C, and fungal burden was expressed as the ratio of colony forming units (CFUs) to the organ weight. For histopathological analysis, organs were fixed in 4% paraformaldehyde, embedded with paraffin wax and stained with periodic acid-Schiff (PAS).

### Mouse model of oral candidiasis

The murine model of oral candidiasis was established according to a previously described method [40], [41]. One day before infection, C57BL/6 mice were immunosuppressed by subcutaneous injection of two doses of prednisolone (100 mg/kg body weight), and the antibiotic tetracycline hydrochloride (0.83 mg/ml) was administered in the drinking water. Immediately before infection, the mice were anesthetised with 50 µl chlorpromazine chloride (2 mg/ml) via intramuscular injection on each femur. The oral cavities of the anesthetised mice were swabbed with a sterilised cotton swab that had been dipped in a cell suspension of *C. albicans* SC5314 (2.5×10^7^ CFUs/ml; from cultures grown overnight at 37°C on SDA). Subsequently, 10 µl SM21 (200 µg/ml; n = 3), 10 µl nystatin (10 µg/ml; n = 3) or 10 µl PBS (as a control; n = 3) was administered to the mice by pipetting into their mouths; this was done five times, at 3, 6, 12, 24 and 36 h after inoculation. The mice were sacrificed 72 h after infection and were immediately observed macroscopically for tongue lesions. The tongue lesion degree was evaluated using a scoring system of 0 – 3 [40], with 0 denoting a healthy tongue surface and 3 the most severe stage. The entire oral cavities of the mice were swabbed with sterilised cotton swabs, which were then submerged in 500 µl PBS and vortexed vigorously. The resultant suspension was spiral plated on SDA, and the number of CFUs was counted after incubation at 37°C for 24 h. In addition, the invasion of tongue tissues by *Candida* was evaluated histologically by PAS staining, as described above.

### Propidium iodide uptake assay

Propidium iodide, a membrane-impermeable fluorescent dye that binds to nucleic acids, is widely used to differentiate cells that have damaged plasma membranes from healthy ones [42], [43]. To evaluate the effect of SM21 on the fungal plasma membrane, *C. albicans* SC5314 cells (approximately 1×10^3^ CFUs/ml) were obtained from logarithmic phase cultures and suspended in RPMI 1640 medium, as described above. The cells were then exposed to sub-MIC of SM21 (0.1 µg/ml) for 1 h at 30°C with gentle shaking. Subsequently, cells were harvested, incubated with propidium iodide using a LIVE/DEAD BacLight Viability Kit (Invitrogen) for 15 min, and then observed by confocal microscopy (Olympus FluoView FV1000).

### Statistical analysis

One-way Analysis of Variance (ANOVA) was employed to evaluate the mean difference between the control and the test groups. Statistical significance of the data was analyzed using SPSS software package (SPSS version 20; SPSS Inc, Chicago, IL, USA). Data was considered significant if p-values are less than 0.05.

## Results

### Discovery of the lead compound

**Figure 2:**
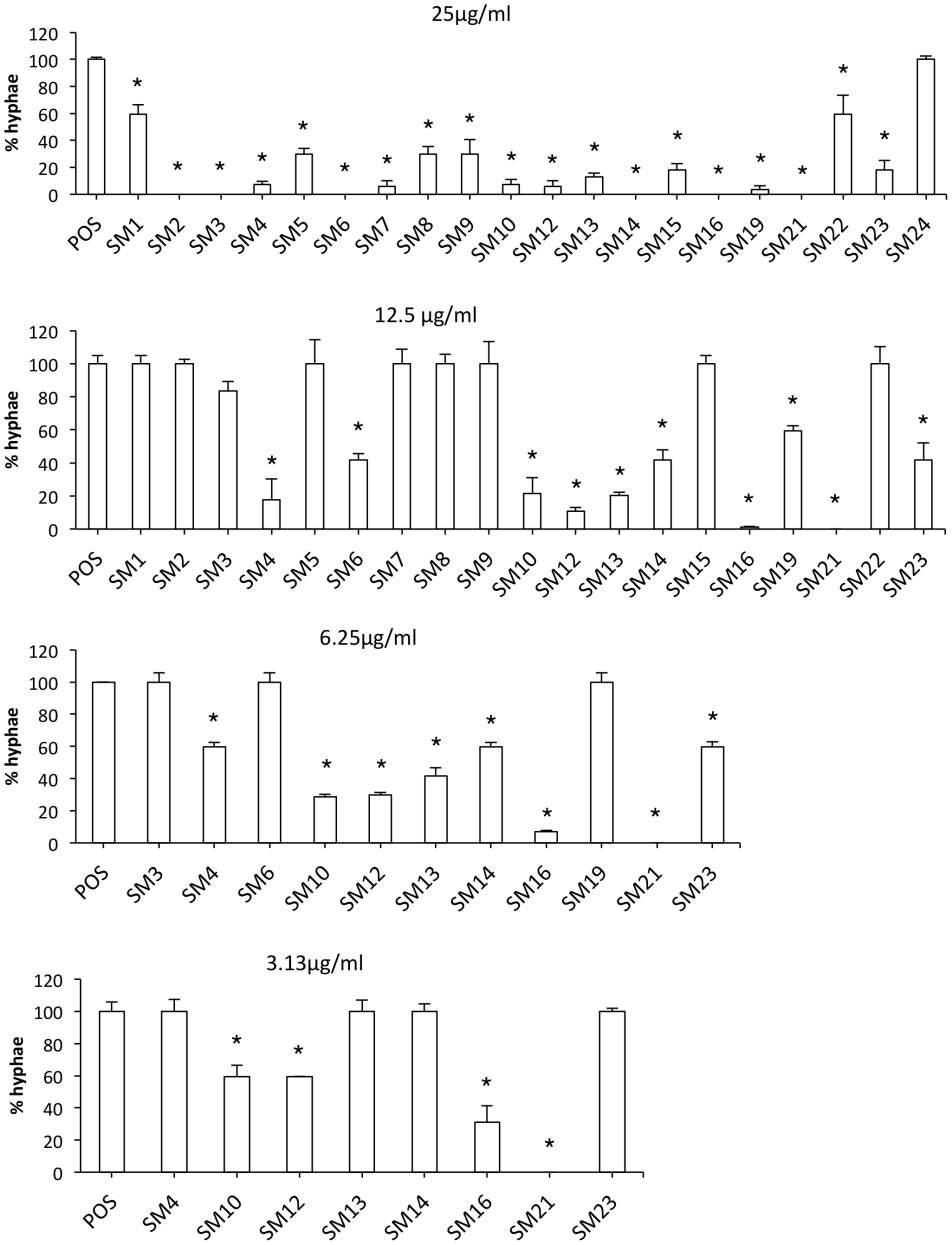
Dose-dependent Y-H inhibition of *C. albicans* SC5314 by the 20 primary hits under hyphal-inducing conditions. Cell morphology of *C. albicans* SC5314 was observed by light microscopy after incubation in the presence of different concentrations of the 20 primary hits under hyphal-inducing conditions (Lee's medium and incubation at 37°C). The degree of Y-H inhibition was quantified by calculating the percentage of hyphal cells in one hundred cells in a single sample. The average percentage of hyphae in the treated samples was normalised to the average percentage of hyphae in the positive control (taken as 100%). Starting with the highest concentration, those small molecules showing no Y-H inhibition were progressively eliminated in subsequent tests, which used lower concentrations. The assay identified eight potent Y-H inhibiting small molecules. The standard deviations are shown for each sample, and asteriks indicate p-value <0.05.

**Figure 3:**
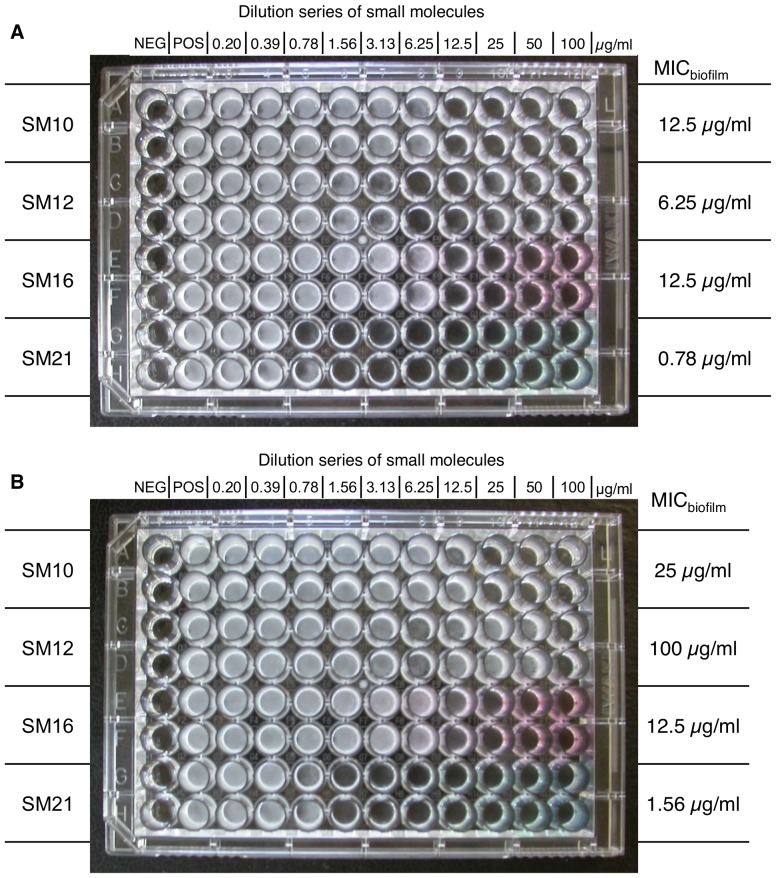
Anti-biofilm properties of SM10, SM12, SM16 and SM21. The small molecules were added to the *C. albicans* SC5314 biofilm (A) before and after the adhesion phase; (B) after the adhesion phase; and incubated in 37°C for 24 h. The MIC_biofilm_ was determined as the concentration where the viability of the biofilm was reduced by 50% as compared with the positive control. SM21 had the lowest MIC_biofilm_ among the four hits in both cases, indicating its potent anti-biofilm property. NEG, negative control; POS, positive control.

**Figure 4:**
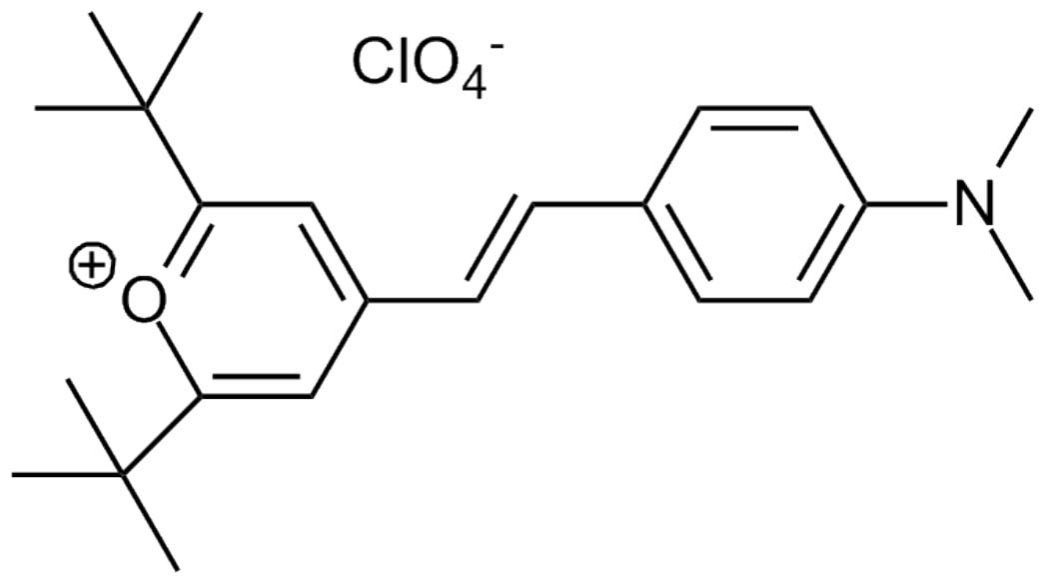
Structure of SM21. The molecular weight of SM21 (ChemBridge ID# 6633321) is 438 g/mol.

To obtain small molecule inhibitors of *Candida* spp., we first screened 50,240 small molecules for inhibitors of the Y-H transition in *C. albicans*, and 20 active compounds were identified. Of these, eight compounds displayed strong dose-dependent Y-H inhibition under hypha-inducing conditions for the reference strain *C. albicans* SC5314 and ten clinical isolates of *C. albicans* (Figure 2). Of these eight compounds, four (SM10, SM12, SM16 and SM21) formed inhibition zones in disk diffusion assays using the standard reference strain *C. albicans* ATCC 90028, indicating fungicidal activity (The result of SM21 is shown in Figure S1). In addition, SM21 had the most potent anti-biofilm activity out of the four compounds (Figure 3). Therefore, SM21 (molecular weight = 438 g/mol, ChemBridge ID# 6633321), which had not been previously identified as an antifungal agent, was chosen for further study (Figure 4).

### SM21 is a potent Y-H inhibitor

**Figure 5:**
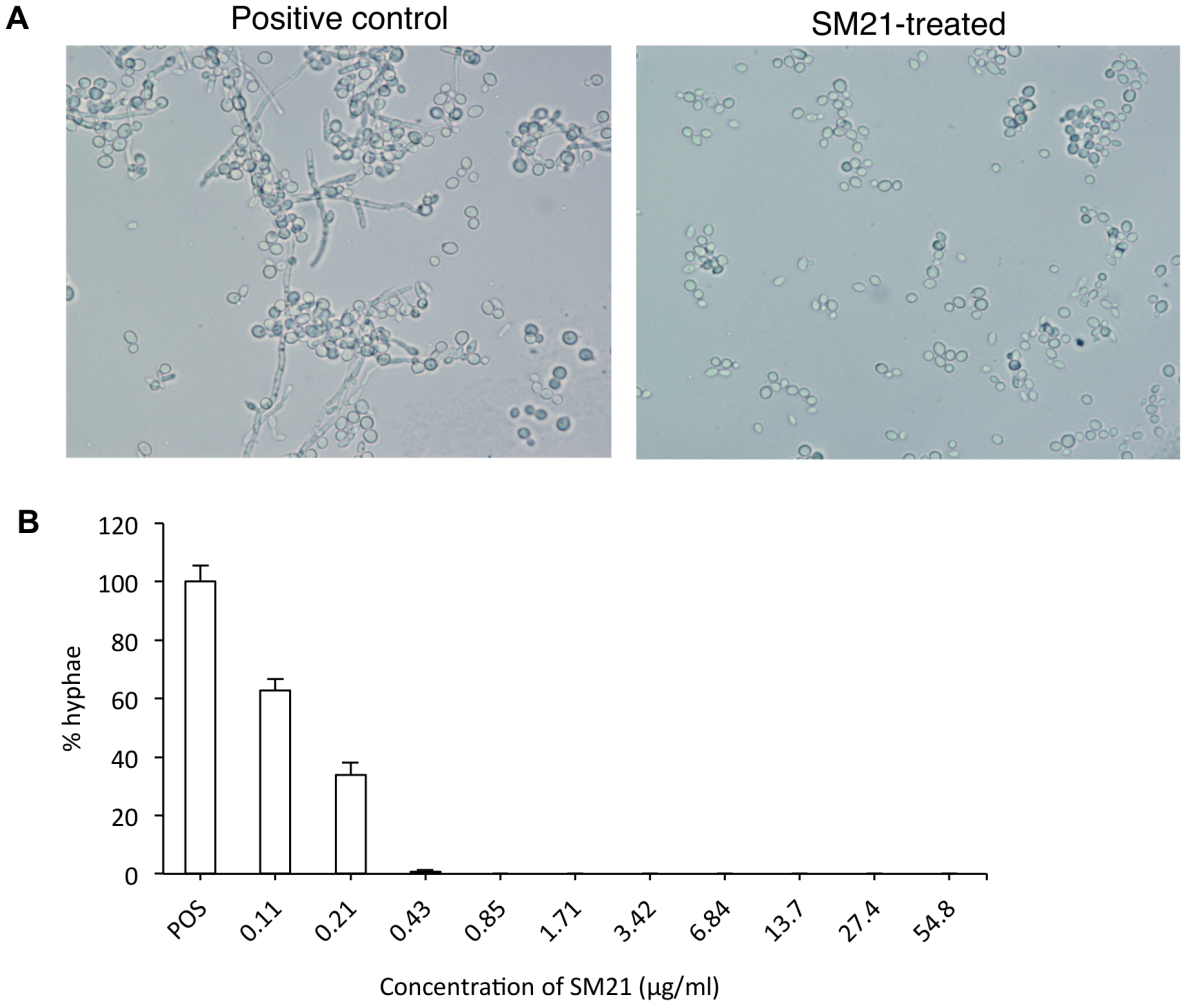
Y-H inhibition by SM21. *C. albicans* cell morphology was observed by light microscopy after incubation in the presence of SM21. (A) SM21-mediated Y-H inhibition against strain CL1, a *C. albicans* clinical isolate. Under hyphal inducing conditions, hyphae could be observed in the positive control (no SM21), whereas, no hyphae were observed in the SM21-treated samples. (B) Dose-dependent effect of SM21 on Y-H inhibition of *C. albicans* SC5314 (1×10^6^ CFUs/ml). POS, positive control (no SM21). The degree of Y-H inhibition of each concentration was quantified by calculating the mean percentage of hyphal cells in one hundred cells in quadruplicate. The average percentage of hyphae in the treated samples was normalised to the average percentage of hyphae in the positive control (taken as 100%). MIC_Y-H_ of SM21 was taken at 0.43 µg/ml, where only minimal amount of hyphae was observed. mean differences between the tests and control were all statistically significant (p-value <0.05).

To further examine the Y-H inhibition of SM21, the phenotype of a panel of *Candida* isolates, including reference strain *C. albicans* SC5314 and ten *C. albicans* clinical isolates of oral origins was observed under robust hypha-inducing condition in the presence of SM21. In the control, where SM21 was not added, extensive hyphae could be observed after 24-h incubation. In contrast, only yeast cells, and not hyphae, could be observed in samples laced with SM21 of concentration 0.43 µg/ml or higher (Figure 5A). The MIC_Y-H_ of SM21 was 0.43 µg/ml (equivalent to 0.98 µM) (for 1×10^6^ CFUs/ml *C. albicans* SC5314; Figure 5B), which was similar to the average MIC_Y-H_ for ten clinical isolates (0.5 µg/ml) (Table 1). Furthermore, the MIC_Y-H_ was directly proportional to the cell density (Table 1).

**Table 1:** Relationship between *C. albicans* cell density and MIC_Y-H_ of SM21.

Cell density	MIC_Y-H_	
(CFUs/ml)	µg/ml	µM
10^4^	0.125	0.29
10^6^	0.5	1.14
10^7^	1	2.28
10^8^	10	22.8

Results are the mean values of three independent experiments, performed in duplicate for each of the ten clinical *C. albicans* isolates CL1 – CL10.

**Figure 6:**
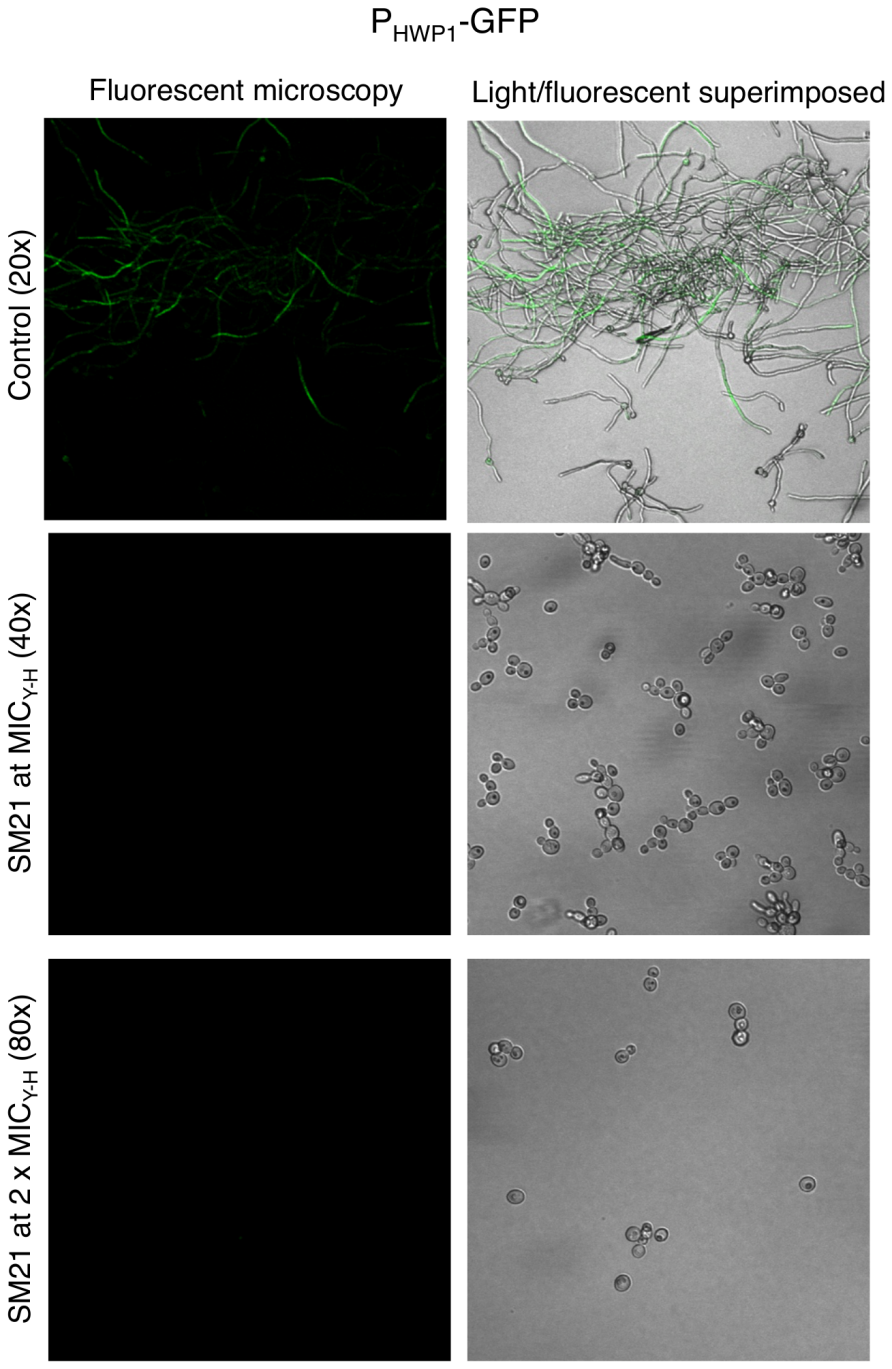
Inhibition of *HWP1* expression by SM21. The inhibition of *HWP1* expression of SM21 was examined by *C. albicans* strain P*_HWP1_*-GFP at MIC_Y-H_ (0.43 µg/ml) and 2×MIC_Y-H_ (0.86 µg/ml). Fluorescence, which indicated GFP expression under the control of the *HWP1* promoter, was observed by confocal microscopy. In the control, multiple hyphae layers were observed under hypha-inducing conditions, however, only the fluorescence from the top layer could be captured by the confocal microscopy. After treatment with SM21 at MIC_Y-H_ or 2×MIC_Y-H_, no trace of fluorescence was detected.

In addition, we used a *C. albicans* P*_HWP1_*-*GFP* strain to test whether SM21 affected *HWP1* expression. GFP was expressed in candidal hyphae under untreated conditions (as observed by confocal fluorescence microscopy; Figure 6). By contrast, no trace of fluorescence could be detected from the SM21-treated cells at both MIC_Y-H_ and 2×MIC_Y-H_, suggesting that SM21 influenced *HWP1* expression.

### SM21 is active against fungi but not bacteria

**Figure 7:**
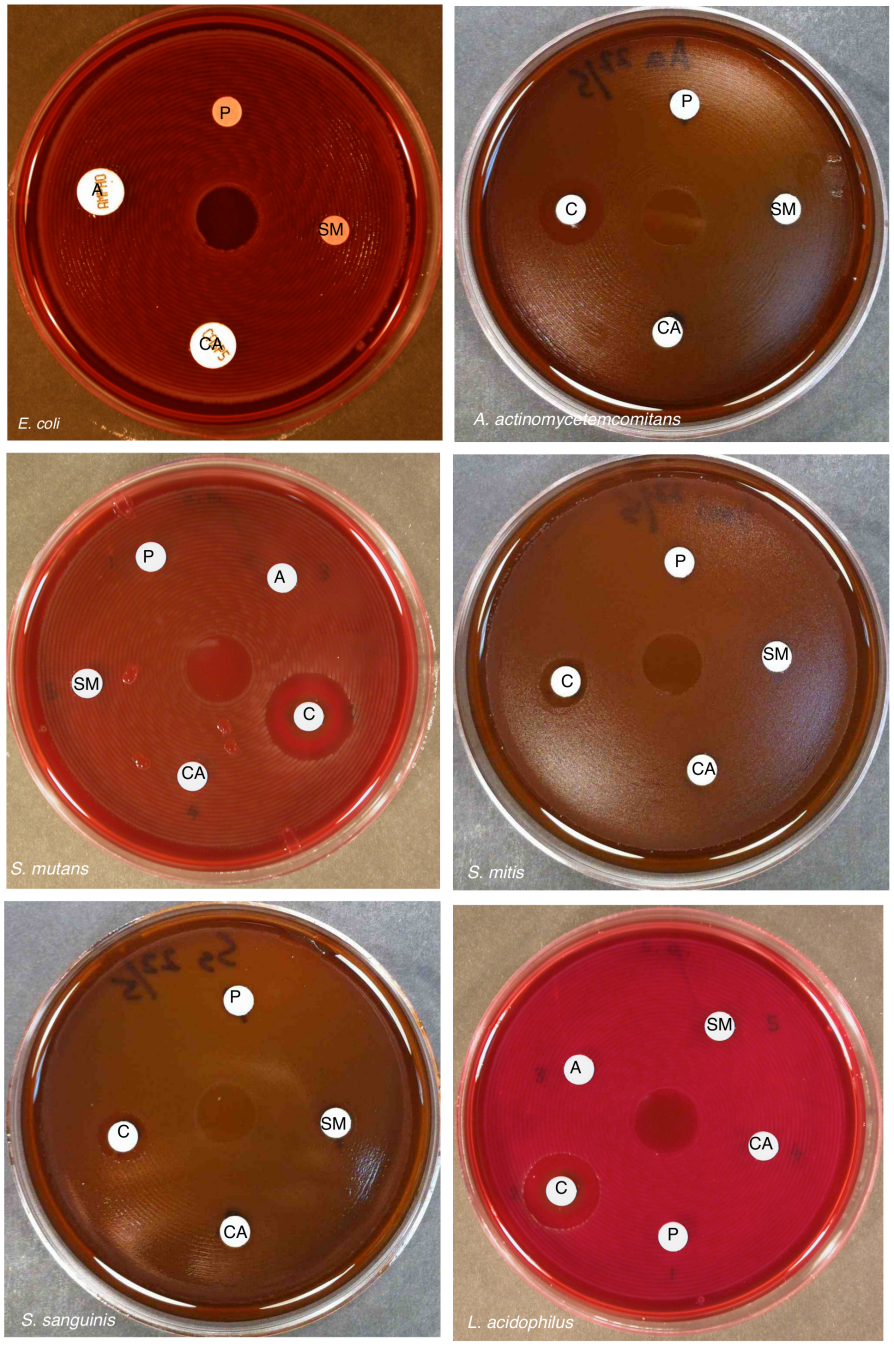
Effect of SM21 on several bacterial species. Disk diffusion assays revealed that SM21 was harmless to *E. coli*, *A. actinomycetemcomitans*, *S. mutans*, *S. mitis*, *S. sanguinis*, and *L. acidophilus*. P – PBS, C – chlorhexidine (0.2%), CA – caspofungin (5 µg), A – amphotericin B (10 µg), SM – SM21 (2 µg).

An ideal antifungal agent should be active against fungal species but not against bacteria. The antifungal activity of SM21 was exhibited by disk diffusion assay and broth microdilution assay. SM21 formed inhibition zones with *C. albicans* SC5314, *C. albicans* ATCC 90028 and all of the 148 clinical isolates tested. The average diameter of the inhibition zones of SM21 against these isolates was 27.7 mm (range = 25 – 31 mm) (The growth inhibition zones of SM21 against *C. albicans* SC5314 and *C. albicans* ATCC 90028 were shown in Figure S1.). For the broth microdilution assay, the MIC of SM21 against *C. albicans* ATCC 90028 was found to be 0.2 µg/ml, which was similar to that of the conventional antifungal amphotericin B, suggesting a comparable potency (Table 2). The MIC of SM21 for a wide range of *Candida* spp. (including clinical isolates) and other fungal species (except *A. fumigatus*) ranged from 0.2 to 1.6 µg/ml (Table 2). Lower susceptibility to SM21 was noted with the fungus *A. fumigatus* (MIC = 6.25 µg/ml), as was also observed for amphotericin B (MIC = 1.56 µg/ml). SM21 did not inhibit the growth of any of the six bacterial species evaluated in disk diffusion assay, implying the fungal specificity of the novel molecule (Figure 7).

**Table 2:** MICs of SM21 and amphotericin B against a range of *Candida* and other fungal species.

	MIC (µg/ml)	
Fungal strain	SM21	AMB
*C. albicans* ATCC90028	0.2	0.2
*C. albicans* SC5314	0.2	0.2
*C. albicans* CL1	0.2	0.2
*C. albicans* CL2	0.2	0.4
*C. albicans* CL3	1.6	0.4
*C. albicans* CL3	1.6	0.4
*C. albicans* CL4	0.8	0.2
*C. albicans* CL5	1.6	0.2
*C. albicans* CL6	1.6	0.2
*C. albicans* CL7	0.8	0.2
*C. albicans* CL8	0.8	0.4
*C. albicans* CL9	1.6	0.4
*C. albicans* CL10	1.6	0.2
*C. glabrata* ATCC90030	1.6	0.4
*C. krusei* ATCC6258	0.4	0.4
*C. tropicalis* ATCC13803	0.8	0.8
*C. parapsilosis* ATCC22019	1.6	0.8
*C. neoformans*	0.4	0.4
*A. fumigatus*	6.25	1.56
*P. marneffei*	0.2	0.05

AMB – amphotericin B. The MICs of SM21 against the *Candida* isolates tested ranged from 0.2 – 1.6 µg/ml, while the MICs of amphotericin B ranged from 0.2 – 0.8 µg/ml. The similar range of MICs suggested the comparable potency of SM21 and amphotericin B. For the other fungal species, lower susceptibility of SM21 against *A. fumigatus* was observed. Such trend was also observed for amphotericin B.

### SM21 inhibits biofilm formation

**Figure 8:**
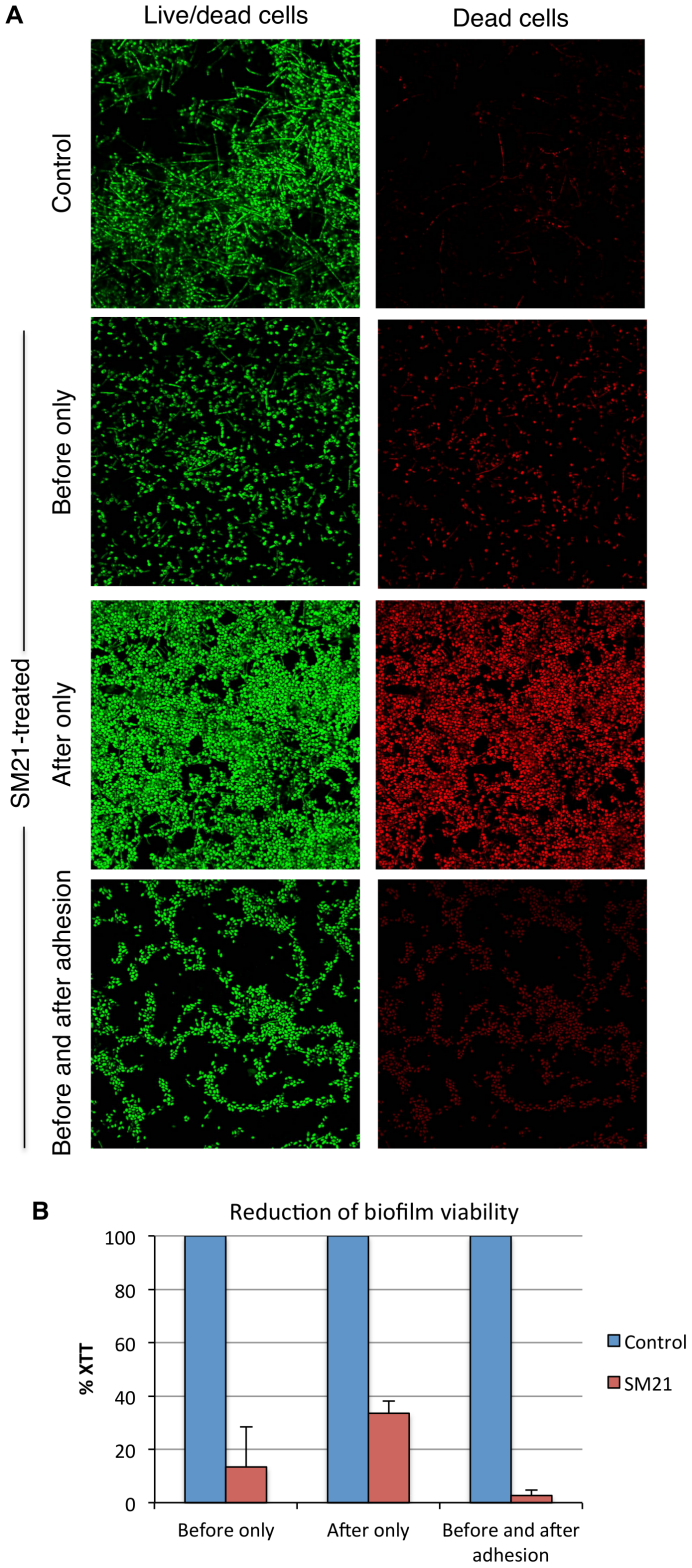
Effect of SM21 on *C. albicans* biofilm formation on denture acrylic. (A) Confocal images of *C. albicans* biofilm after treatment with SM21 and staining with fluorescent labels that distinguish between live and dead cells. All cells were labeled with the green fluorescence, while only the dead cells were labeled with red fluorescence. (B) Biofilm cell viability, quantified by XTT reduction assay, was reduced by 85% and 66%, respectively, when SM21 was added before or after the adhesion phase. The reduced biofilm viability was maximised (97%) when SM21 was added both before and after the adhesion phase. The standard deviations of each sample are shown in the graph, and all the mean differences between the control and test (SM21) were statistically significant (p-value<0.05).

We then assessed the activity of SM21 against *Candida* biofilm formation. We first used a microtitre-plate biofilm model in which SM21 was added to the 24-h and 48-h biofilm of *C. albicans*, *C. glabrata* and *C. krusei*. SM21 demonstrated a lower MIC_biofilm_ than amphotericin B and caspofungin for both 24-h and 48-h biofilm (Table 3). In an *in vitro* model of denture stomatitis, SM21 effectively prevented candidal adhesion and biofilm development on denture acrylic surfaces (Figure 8A). In this model, biofilm viability was reduced by 85%, 66% and 97%, when SM21 was added before, after, or both before and after the 1.5 h adhesion phase respectively (Figure 8B). Summarized from the two assays, SM21 was effective against mature *Candida* biofilm and inhibited biofilm formation on denture acrylic surfaces.

**Table 3:** MIC_biofilm_ of SM21 against three different *Candida* species.

	MIC (µg/ml)					
*Candida* species	24-h biofilm			48-h biofilm		
	SM21	AMB	CASP	SM21	AMB	CASP
***C. albicans***	3.2	32	50	25	32	100
***C. glabrata***	3.2	16	50	25	16	100
***C. krusei***	3.2	32	50	25	32	100

*Candida* biofilms of 24-h or 48-h were incubated with serial dilution of SM21 for 24 h. Thereafter, the MIC_biofilm_ of SM21 was determined as the concentration at which the cell viability of the biofilm was reduced by 50% as compared with the untreated control. (AMB – amphotericin B, CASP – caspofungin.)

### SM21 is active against antifungal-resistant *Candida* spp.

**Figure 9:**
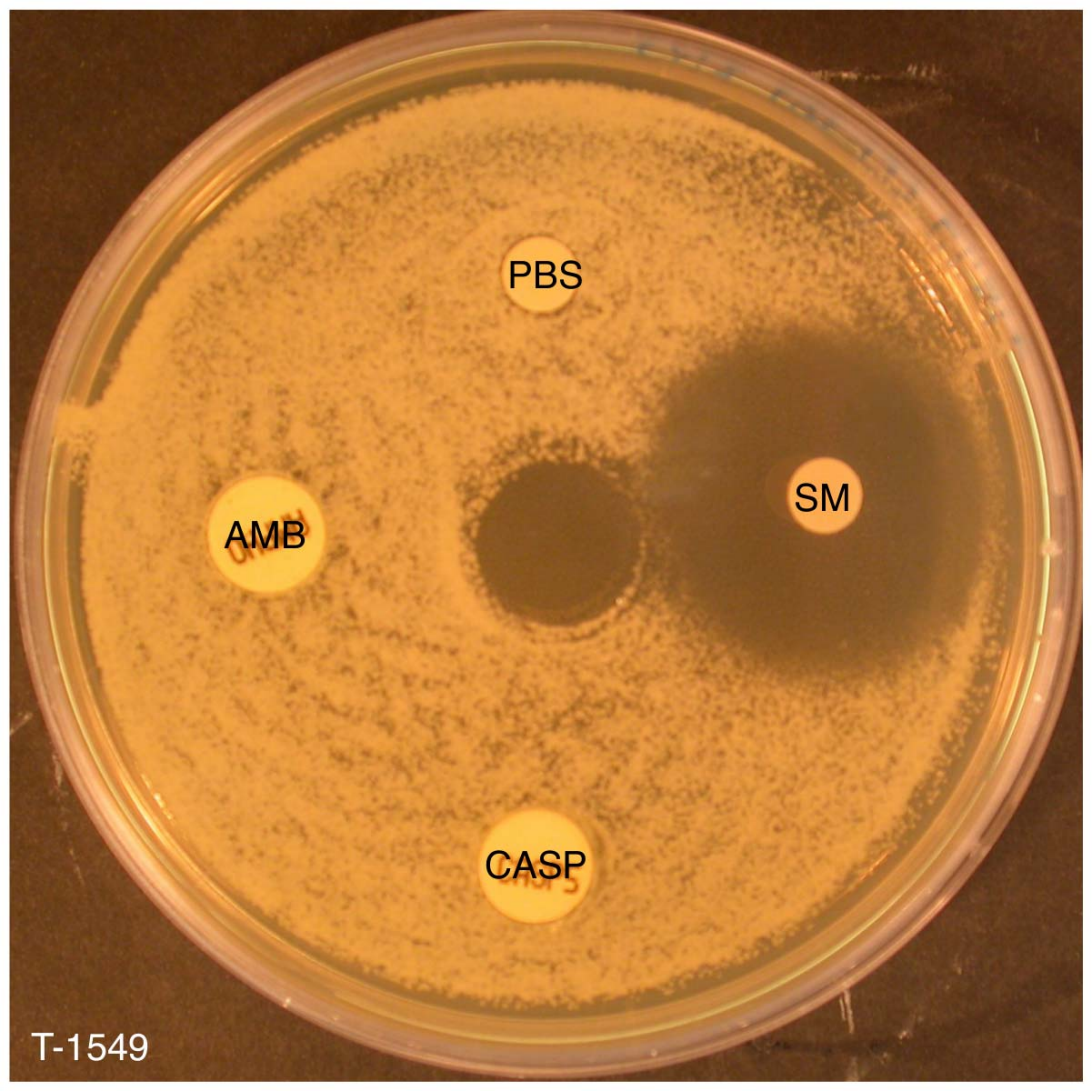
Effect of SM21 on a multidrug-resistant isolate. SM21 (SM) produced a clear inhibition zone in a disk diffusion assay against T-1549, a *C. guilliermondii* strain with multidrug resistance to amphotericin B (AMB), caspofungin (CASP) and fluconazole.

The emergence of antifungal-resistant *Candida* strains has led to the urgent need of new antifungal agents. All of the drug-resistant isolates tested were susceptible to SM21. For instance, a clear SM21 inhibition zone was observed for T-1549, a multidrug-resistant *C. guilliermondii* strain (Figure 9). The MIC of SM21 against drug-resistant *Candida* isolates in broth microdilution assays ranged from 0.5 to 1 µg/ml (Table 4), which was marginally higher than the MIC for *C. albicans* ATCC 90028.

**Table 4:** MICs of SM21 for antifungal-resistant *Candida* strains.

Drug-resistant isolates	Antifungal-resistance	MIC (µg/ml)
*C. albicans* T1675	F,I	0.5
*C. parapsilosis* T1677	C,F,I,K,V	1
*C. parapsilosis* T1565	C,I	1
*C. parapsilosis* T1545	C	1
*C. parapsilosis* T1688	I	0.5
*C. glabrata* T1585	F,I,K,V	1
*C. glabrata* T1672	I,K	0.5
*C. glabrata* T1570	I	0.5
*C. tropicalis* T1427	I	1
*C. tropicalis* T789	I	1
*C. tropicalis* T1148	I	0.5
*C. krusei* T1472	F,I,K	1
*C. krusei* T1266	F	0.5
*C. krusei* T1562	F	1
*C. krusei* T1266	F	1
*C. guilliermondii* T1549	A,C,F	1

A – amphotericin B, C – caspofungin, F – fluconazole, I – itraconazole, K – ketoconazole, V – voriconazole.

### SM21 displays low toxicity to human cells

**Figure 10:**
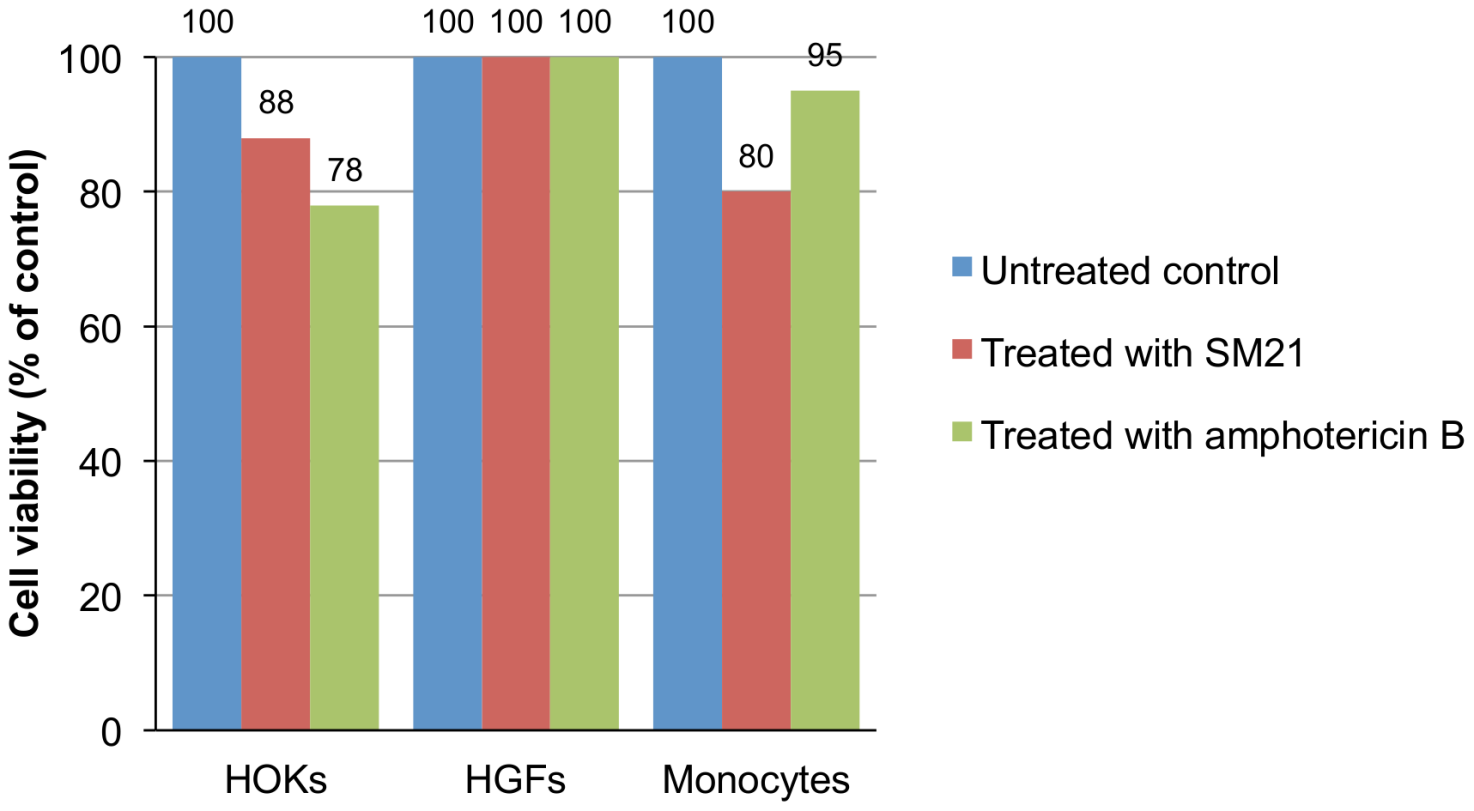
Cytotoxicity of SM21 against three primary cell lines. HOK – human oral keratinocyte, HGF – human gingival fibroblast, AMB – amphotericin B. Both SM21 (0.2 µg/ml) and amphotericin B (0.2 µg/ml) reduced the viability of HOKs and monocytes. SM21 caused a reduction of 12% in cell viability compared with the untreated controls, which is lower than the 22% reduction observed for amphotericin B. Treatment with SM21 or amphotericin B caused, respectively, a 20% or 5% reduction in monocyte viability compared with the controls. Neither SM21 nor amphotericin B affected the viability of HGFs.

Cytotoxicity assays performed in a 96-well plate revealed the CC_50_ of SM21 to be 3.4 µg/ml (7.8 µM) for HOKs. In assays conducted in T-75 flasks, 0.2 µg/ml SM21 reduced the viability of HOKs by 12% compared with the untreated controls, which is lower than the 22% reduction observed for amphotericin B. Treatment with SM21 or amphotericin B caused, respectively, a 20% or 5% reduction in monocyte viability compared with the controls. Neither SM21 nor amphotericin B affected the viability of HGFs (Figure 10).

### SM21 prevents candidal invasion of human cells in vitro

**Figure 11:**
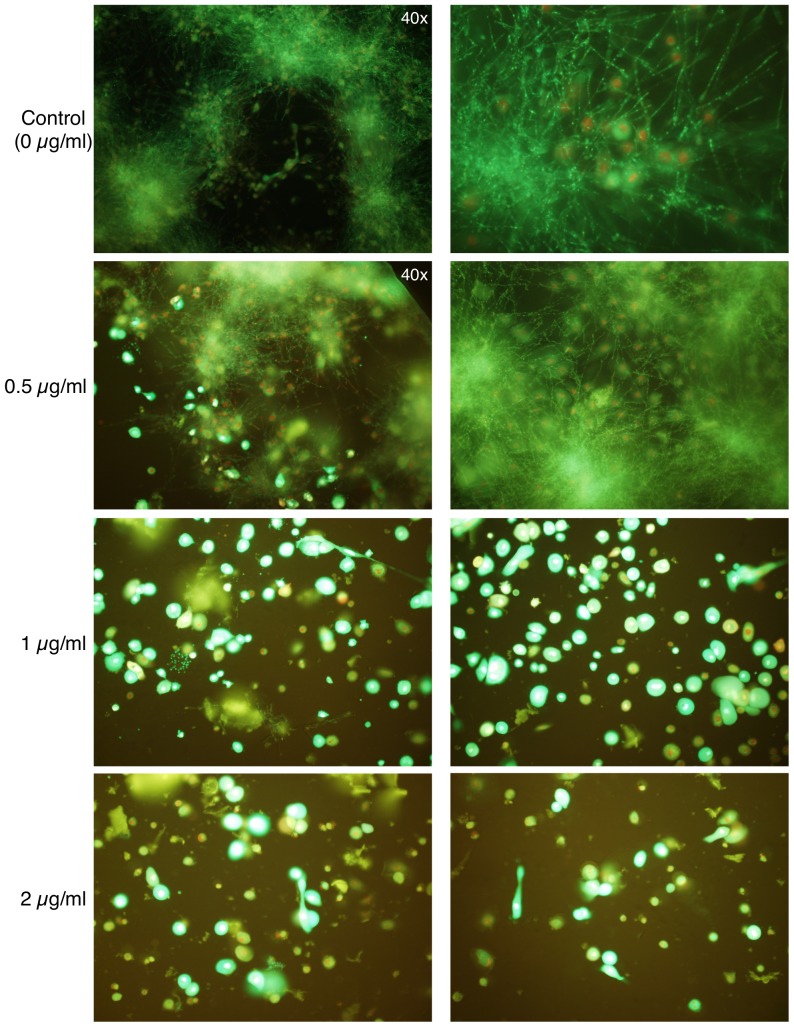
Effect of SM21 treatment in *Candida*–HOK co-culture model. The viability of co-cultured HOKs and yeast cells in the presence of SM21 was assessed by confocal laser scanning microscopy using fluorescent probes. In the untreated control, many hyphae were observed, and most of the HOKs were dead (the nuclei of the dead cells were labeled with red fluorescence). Samples containing 0.5 µg/ml SM21 still included many hyphae, but they contained more live HOKs than the untreated control. Samples containing 1 µg/ml or 2 µg/ml SM21 included few yeast cells and no hyphae, and most of the HOKs were alive. Magnification = 20× (unless specified otherwise).

To test whether SM21 was able to prevent *C. albicans* invasion of human cells, a co-culture of HOKs and *C. albicans* SC5314 was exposed to various concentrations of SM21. In the co-culture control (in which no SM21 was present), many hyphae were observed. The hyphal invaded and caused the death of most of the HOKs (Figure 11). At 0.5 µg/ml SM21, *Candida* hyphae could still be observed, however, the proportion of live HOKs increased. At 2 µg/ml and 4 µg/ml SM21, most of the HOKs were alive, and no *Candida* were observed. Therefore, this SM21 concentration was safe to HOKs and was sufficient to prevent *Candida* from invading the human cells.

### SM21 improves survival in a mouse model of systemic candidiasis

**Figure 12:**
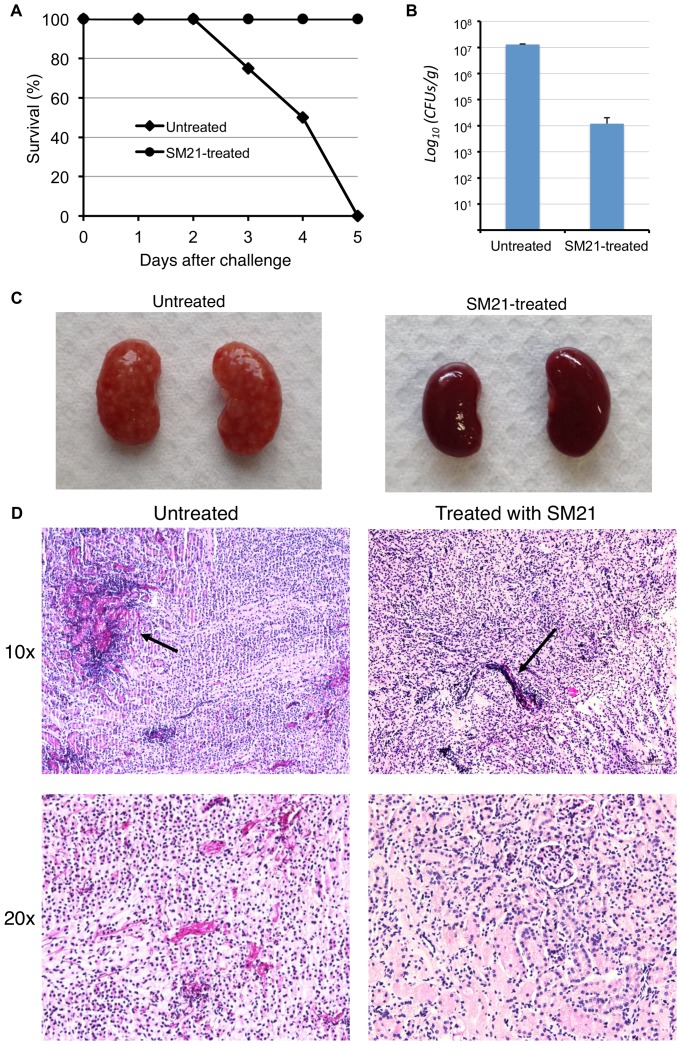
Effect of SM21 treatment on a mouse model of systemic candidiasis. (A) Survival rate of the mice. After the experimental period (day 5), the survival rates for untreated and SM21-treated mice were 0% and 100%, respectively. (B) Fungal burden in the kidneys of the mice (Error bars indicate standard deviation). SM21 significantly reduced the renal fungal burden in the mice by an order of magnitude of 3 (p-value of mean difference <0.05). (C) Surfaces of the kidneys of untreated mice were covered with *Candida* lesions, while the kidneys of the SM21-treated mice appeared healthy. (D) PAS staining of the kidneys. *Candida* hyphae (indicated by black arrows) were easily spotted in the kidneys of untreated mice, whereas few were detected in the kidneys of SM21-treated mice.

A mouse model of systemic candidiasis was used to assess the *in vivo* activity of SM21. Infection was established in mice by injecting 100 µl of 1×10^6^ CFUs/ml *C. albicans* SC5314 via tail vein. The test group was treated with 0.01, 0.1, 1 and 10 mg/kg SM21, while control group was treated with PBS. At day 5 (the experimental end point) after infection with *C. albicans*, all of the untreated mice with systemic candidiasis had died, whereas all of the SM21-treated mice were alive (Figure 12A). The fungal burden in the kidneys was significantly lower in the SM21-treated mice than in the untreated ones (Figure 12B). Moreover, white *Candida* lesions were visible covering the surfaces of the kidneys of the untreated mice, while the kidneys of the SM21-treated mice appeared healthy (Figure 12C). Clusters of *Candida* hyphae were easily identified in PAS-stained kidneys of the untreated mice, whereas very few *Candida* cells were observed in the kidney parenchyma of the SM21-treated mice (Figure 12D). These results indicated that SM21 was effective in preventing systemic spread and invasion of *Candida* in the mouse model.

### SM21 reduces tongue lesions in a mouse model of oral candidiasis

**Figure 13:**
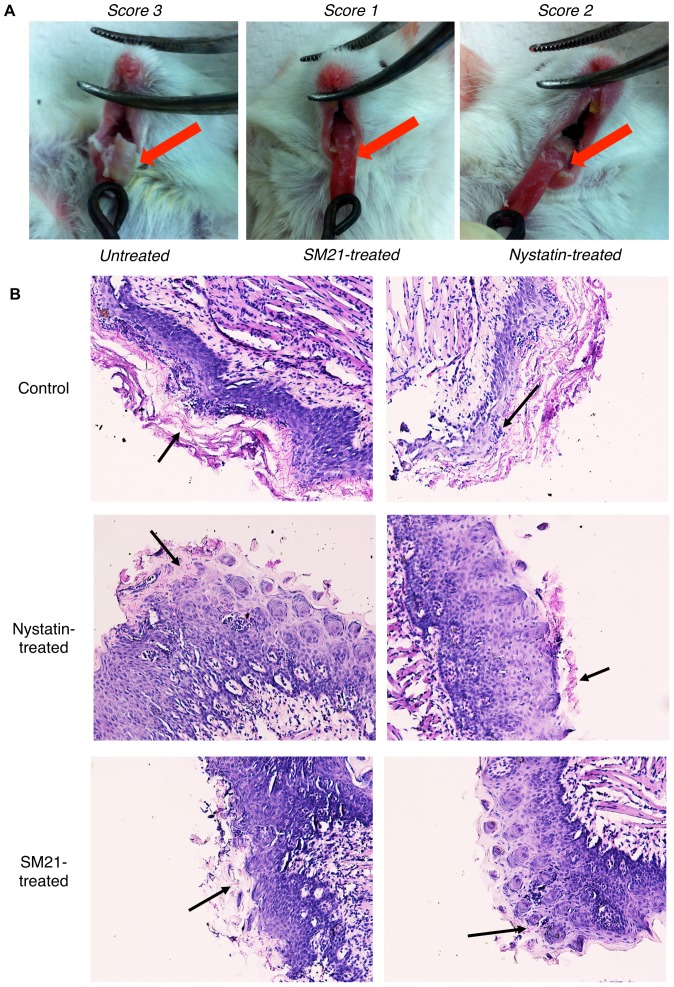
Effect of SM21 treatment on a mouse model of oral candidiasis. (A) The degree of tongue lesions in the oral candidiasis mouse model was evaluated by a scoring system of 0 – 3 (0 denotes healthy tongue surface and 3 denotes the most severe stage). A thick oral thrush (score = 3) was observed in untreated mice. Nystatin-treated mice displayed less oral thrush on the tongue surface than the untreated controls, but a considerable amount of oral thrush was observed at the back of the tongue (score = 2). SM21-treated mice displayed the least severe tongue lesions (score = 1). (B) PAS staining of tongue sections. Abundant hyphae (indicated by black arrows) covered most of the tongue surface in the untreated mice, whereas comparatively fewer hyphae were observed in nystatin- and SM21-treated mice.

We then assessed the effects of SM21 and nystatin treatments in a mouse model of oral candidiasis established by inoculating *C. albicans* SC5314 (2.5×10^7^ CFUs/ml) in the oral cavities of the mice. Treatment was given to the mice at five time intervals. Immediately after sacrifice (72 h after inoculation), scores were assigned for tongue lesions from 0 (healthy tongue surface) to 3 (most severe stage). Thick oral thrush was observed in the untreated mice (score = 3; Figure 13A). Tongue lesions in the nystatin-treated mice were less severe than in the untreated controls, although considerable oral thrush was noted at the back of the tongue (score = 2). Compared with the other two groups, SM21-treated mice displayed the least severe tongue lesions (score = 1). Moreover, *Candida* cell counts from tongue swabs of the SM21-treated mice (4.5×10^2^ CFUs/ml) were consistently lower than those of the control mice (4.8×10^3^ CFUs/ml) and the nystatin-treated animals (6.0×10^2^ CFUs/ml). Numerous candidal hyphae were noted in PAS-stained tongue sections of the untreated mice, whereas less profuse candidal growth was observed in samples from the mice treated with SM21 or nystatin (Figure 13B).

### SM21 affects permeability of the fungal cell membrane

**Figure 14:**
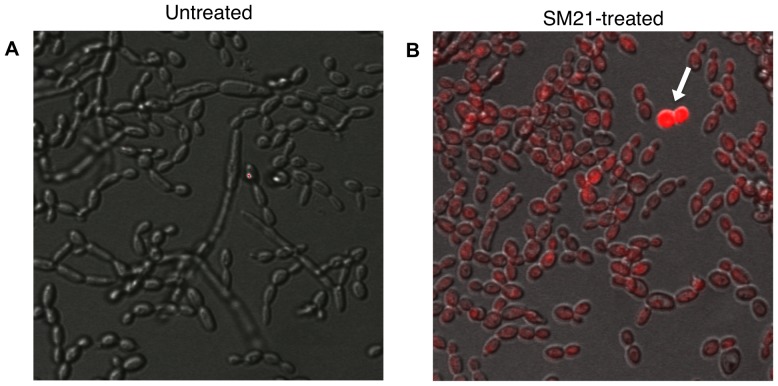
Confocal microscopic analysis of propidium-iodide uptake assays. (A) Untreated control samples showed hyphal elements of live *C. albicans* cells. (B) *C. albicans* treated with sub-MIC of SM21 (0.1 µg/ml) showed propidium iodide uptake (labeled by red fluorescence), indicating cell membrane damage. Some of the cells (denoted by white arrow) were non-viable.

To shed some light on the mechanism of action of SM21, we performed propidium-iodide uptake assays. *Candida* exposed to sub-MIC of SM21 (0.1 µg/ml) displayed abundant propidium iodide uptake, indicating cell membrane damage (Figure 14). Therefore, the molecular target of SM21 might be a component of the cell membrane.

## Discussion

A handful of small molecules have been reported to possess activity against *C. albicans* [29], [44]–[47]. However, the therapeutic potential of these small molecules is unclear, because they have been evaluated mainly for the ability to inhibit *C. albicans* virulence properties such as Y-H transition or biofilm formation *in vitro*. It has been hypothesised that arresting *Candida* in the unicellular yeast form would result in reduced virulence. This hypothesis is supported by the finding that non-filamentous *C. albicans* mutants are not virulent in a mouse infection model [48], however, this is only relevant to *C. albicans*. Some NAC species (which do not form true hyphae) are also virulent. Moreover, the strategy of inhibiting virulence factors could facilitate the emergence of resistant strains because it only inhibits the growth of virulent strains but does not kill them. Complete elimination of pathogen is clearly a safer option; therefore, fungicidal drugs are preferred.

For all of the above reasons, we set out to identify new antifungal compounds with both fungicidal and anti-virulence properties. To this end, we first screened for Y-H inhibitors a library of 50,240 small molecules. It should be noted that the Y-H inhibitor screening assay selects for molecules that block the change from the yeast form of *C. albicans* to the hyphal form, as assessed by morphology. Therefore, this assay does not discriminate between molecules that inhibit the growth of *C. albicans*, which would also prevent the Y-H transition, and true inhibitors of the Y-H transition. Moreover, conventional antifungal agents such as fluconazole, amphotericin B and 5-fluorocytosine, whose mechanisms are not directly related to Y-H inhibition, also inhibit the formation of hyphae [49]. Nevertheless, this ambiguity of the screening assay was favourable for increasing the hit rate in our initial screening.

After several *in vitro* analyses of the antifungal properties of 20 small molecules identified by our initial screening, we found that one of them, SM21, is a potent Y-H inhibitor that is effective at low concentrations. Among the Y-H inhibitors identified in the study by Midkiff et al, the lowest MIC_Y-H_ is 20 µM for 1×10^4^ cells [46], which is much higher than the SM21 MIC_Y-H_ (0.98 µM for 1×10^6^ CFUs/ml). The number of *Candida* decreased after SM21 treatment *in vitro*, suggesting that this compound is fungicidal. Fungicidal drugs inhibit Y-H transition at sub-MIC, whereas fungistatic drugs inhibit Y-H transition only at much higher concentrations than their MIC [50]. It has been suggested that fungicidal drugs inhibit hyphal development and the budding process, whereas fungistatic drugs inhibit only the budding process [50].

The *in vitro* antifungal activity of SM21 is comparable to that of commonly used antifungal drugs such as amphotericin B (a polyene). In disk diffusion assays, SM21 forms clear inhibition zones that resemble those produced by polyenes and echinocandins, and unlike the partial inhibition zones formed by azoles, for which trailing endpoint is observed [51]. The MIC of SM21 in broth microdilution assays for a wide range of *C. albicans* strains (0.2 – 1.6 µg/ml) is similar to the MIC of amphotericin B, which has been considered to be a gold standard of antifungal agents [52]. Moreover, the activity of SM21 against *C. albicans* biofilms grown on microtitre plates is similar or more potent than that of amphotericin B or caspofungin (Table 3). The MIC_biofilm_ of SM21 increases as the biofilm matures, a common phenomenon among antifungal agents [53].

Attachment to mucosal tissue and biofilm formation are the basis of mucosal candidiases such as oral candidiasis and *Candida*-associated denture stomatitis [4], [9]. SM21 is effective at reducing candidal attachment to denture acrylic surfaces and inhibiting biofilm development in an *in vitro* model of denture stomatitis. These promising data indicate that SM21 might have potential as a denture disinfectant or as the active ingredient in an oral drug delivery system.

We also analysed the *in vivo* effects of SM21 treatment on mouse models of oral and systemic candidiasis. We showed that oral rinses containing 200 µg/ml SM21 significantly reduced tongue lesions in the oral candidiasis mouse model. Moreover, the efficacy of SM21 was better than that of nystatin, which is the most commonly used antifungal for oral candidiasis [4]. In particular, oral thrush was observed at the back of the tongues in nystatin-treated mice (indicating oropharyngeal candidiasis) but not in SM21-treated animals. However, very little is known about the pharmacokinetics and pharmacodynamics of nystatin in murine models of oral candidiasis, and therefore the concentration used in our experiments (10 µg/ml) might not be high enough to produce an optimal outcome. In addition, the duration of an oral rinse cannot be controlled in a mouse model.

We evaluated SM21 toxicity against human cell lines *in vitro*. In contrast to bacteria, the cells of fungi and humans are eukaryotic and, therefore, very similar. This similarity is a major hurdle in antifungal development, which requires drug targets to be fungus-specific to avoid undesirable toxicity [54]. We found that the CC_50_ of SM21 for HOKs is 7.8 µM (3.4 µg/ml) in a 96-well plate assay. Although this value is lower than the minimum CC_50_ (16 µM) reported for 14 antifungal compounds that were identified by LaFleur et al (tested against human fibroblasts in 96-well plates; [45]), the CC_50_ of SM21 is still higher than its MIC (0.2 µg/ml), which indicates that there is a range of concentrations at which SM21 could be used as an antifungal agent without causing significant toxicity to human cells. Indeed, no detrimental effects were observed in mice that had been injected with SM21 doses as high as 10 mg/kg twice daily. In addition, SM21 does not seem to have antibacterial properties, indicating that its target is present in fungi but not in bacteria.

We demonstrated that intraperitoneal administration of SM21 is an effective treatment for systemic candidiasis in a mouse model. An SM21 dose of 0.1 mg/kg or higher twice daily beginning 3 h post-infection significantly reduced fungal burden in non-neutropenic mice. The *in vivo* efficacy of SM21 is similar to that of conventional antifungal agents in comparable non-neutropenic mouse models of systemic *C. albicans* candidiasis. For example, the intraperitoneal ED_50_ (50% effective dose) of fluconazole is 4.56 mg/kg when administered to the mice as a single dose 5 h after infection; the animals were sacrificed 24 h later [55]. Intraperitoneal amphotericin B (1 mg/kg dose daily) decreases fungal burden in mouse kidneys from day 1 to day 3 after infection (with treatment beginning 24 h after infection) [56]. In addition, intravenous micafungin treatment (0.125 mg/kg or higher daily dose, starting 1 h after infection for 4 days) prolongs survival of infected mice [57]. However, it is difficult to compare the effective doses from different studies because there is no standard protocol, and differences in the *Candida* strain, inoculum size, mouse strain, mouse immune status and the start and interval of antifungal therapy could interfere with the outcome. In addition, there are differences in the *in vivo* drug efficacy among normal and neutropenic murine models, which may be due to the possible interactions between the drug and host defence mechanisms [56]. Detailed pharmacodynamics-pharmacokinetics studies would need to be performed in the future to determine the time/concentration relationship of the drug at the infection sites and, thus, to generate the dosing regimens that produce the best treatment outcome *in vivo* [58].

SM21 is effective against antifungal-resistant clinical isolates, indicating that its mechanism of action is distinct from those of conventional antifungals. Our preliminary results described here indicated that SM21 could be targeting a component of the fungal cell membrane. Nevertheless, extensive assays are needed to elucidate the mechanism of action of this new drug.

In conclusion, in the present study we have characterised novel antifungal small molecule, SM21 using a comprehensive set of *in vitro* and *in vivo* assays. Our findings support SM21 as a promising compound for development of novel antifungal agent for treating local and systemic candidiasis, which could bring enormous benefit to the patients suffering from this recalcitrant fungal pathogen.

## Supporting Information

Figure S1 **Antifungal susceptibility test (disk diffusion assay) of SM21 against** ***C. albicans*** **SC5314 and** ***C. albicans*** **ATCC 90028.** 21 – SM21, A – amphotericin B, F – fluconazole, K – ketoconazole. Growth inhibition zones were observed for SM21 against the two *C. albicans* strains tested. The growth inhibition zones produced by SM21 were clear, similar to those produced by amphotericin B, but dissimilar to those produced by fluconazole. This phenomenon suggested that SM21 is, like amphotericin B, fungicidal in nature.
(TIF)
