# Supplementary figures and images for: In Vitro and In Vivo Activity of a Novel Antifungal Small Molecule against Candida Infections
Source: PLoS One. 2014 Jan 22;9(1):e85836. doi: 10.1371/journal.pone.0085836 (PMC3899067; doi:10.1371/journal.pone.0085836)

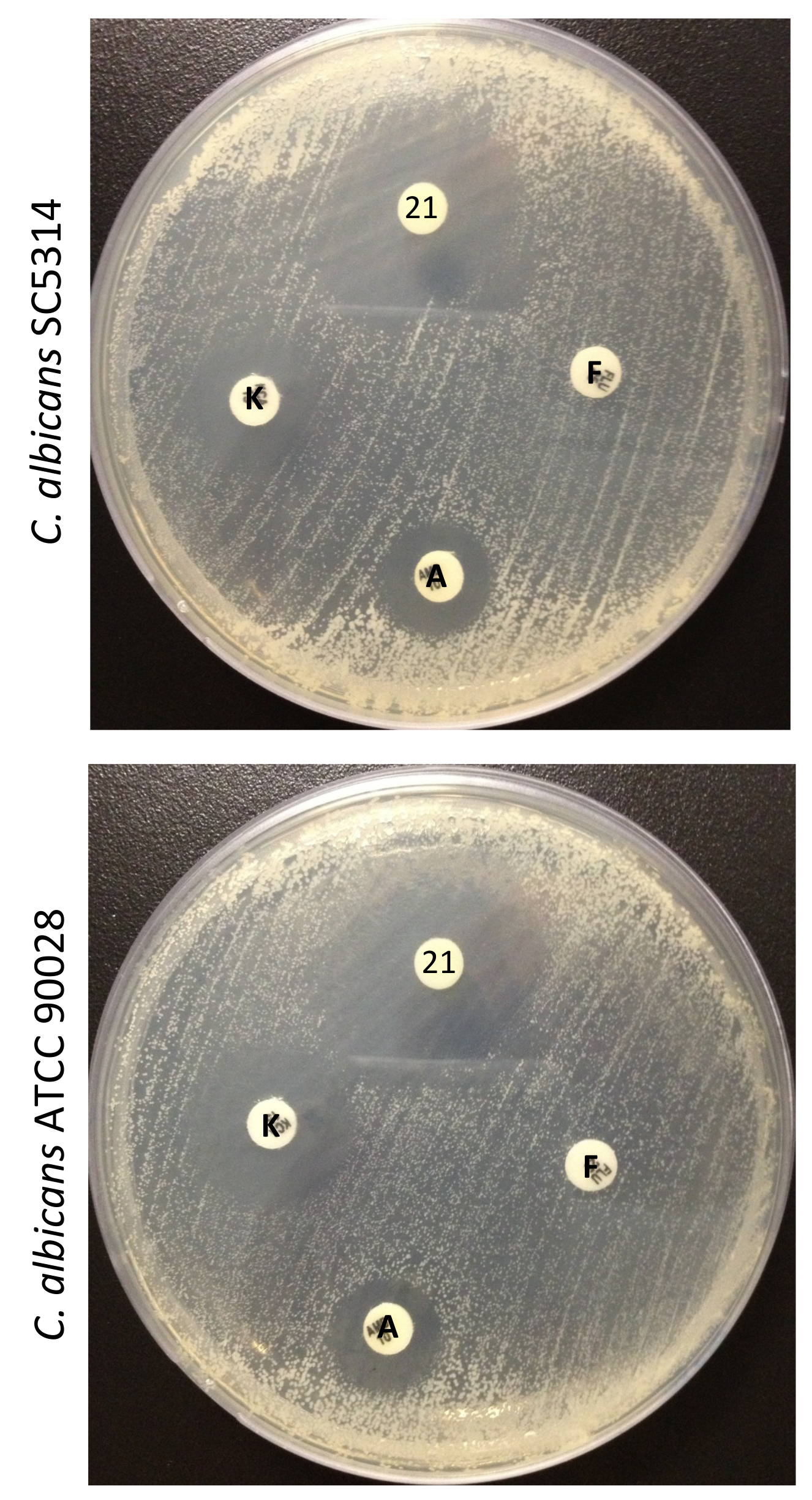

Supplement: Figure S1 — Antifungal susceptibility test (disk diffusion assay) of SM21 against C. albicans SC5314 and C. albicans ATCC 90028. 21 – SM21, A – amphotericin B, F – fluconazole, K – ketoconazole. Growth inhibition zones were observed for SM21 against the two C. albicans strains tested. The growth inhibition zones produced by SM21 were clear, similar to those produced by amphotericin B, but dissimilar to those produced by fluconazole. This phenomenon suggested that SM21 is, like amphotericin B, fungicidal in nature. (TIF) [file pone.0085836.s001.tif]
